# The Efficacy of Programmed Intermittent Epidural Bolus for Postoperative Analgesia after Open Gynecological Surgery: A Randomized Double-Blinded Study

**DOI:** 10.1155/2018/6297247

**Published:** 2018-05-15

**Authors:** Shiho Satomi, Nami Kakuta, Chiaki Murakami, Yoko Sakai, Katsuya Tanaka, Yasuo M. Tsutsumi

**Affiliations:** Department of Anesthesiology, Tokushima University, Kuramoto, Tokushima, Japan

## Abstract

**Background:**

It is well known that the programmed intermittent epidural bolus (PIEB) technique effectively provides epidural anesthesia in labor. This randomized double-blind trial compared the postoperative analgesic efficacy of PIEB with that of continuous epidural infusion (CEI) in patients undergoing gynecological surgery under combined general-epidural anesthesia.

**Methods:**

Patients undergoing open gynecological surgery under combined general-epidural anesthesia were randomized at a 1 : 1 ratio to receive PIEB or CEI. In the PIEB group, the pump delivered 4 mL ropivacaine 0.2% plus fentanyl 2 *μ*g/mL every hour. In the CEI group, the pump delivered the same solution at a rate of 4 mL/h. In both groups, additional 4 mL boluses of ropivacaine 0.2% plus fentanyl 2 *μ*g/mL were provided, when necessary, by patient-controlled epidural analgesia after surgery. The primary outcome was the total ropivacaine dose 40 hours after surgery. The secondary outcomes were the number of PCEA boluses and postoperative pain (evaluated on an 11-point numerical rating scale) 3, 24, and 48 hours after surgery.

**Results:**

In total, 57 patients were randomized (*n* = 28 and 29 in the PIEB and CEI groups, resp.). The two groups differ significantly in terms of the total ropivacaine dose 40 hours after surgery (mean (standard deviation): 155.38 (4.55) versus 159.73 (7.87) mL, *P* = 0.016). Compared to the CEI group, the PIEB group had significantly lower numerical rating scale scores 3 hours (median [lower–upper quartiles]: 0 [0–0.5] versus 3 [0–5.5], *P* = 0.002), 24 hours (1 [0–2] versus 3 [1–4], *P* = 0.003), and 48 hours (1 [0–2] versus 2 [2–3.5], *P* = 0.002) after surgery.

**Conclusion:**

PIEB was better than CEI in terms of providing postoperative analgesia after open gynecological surgery under combined general-epidural anesthesia.

## 1. Introduction

Epidural infusion is an effective neuraxial analgesic technique that has been used to manage postoperative pain for decades [1]. In recent years, the efficacy of the programmed intermittent epidural bolus (PIEB) technique has been well demonstrated for labor analgesia [2–9]. PIEB is an automated method of administering boluses of local anesthetic solution to the epidural space at fixed, scheduled time intervals. PIEB prolongs the duration of analgesia, reduces motor block, lowers the incidence of breakthrough pain, improves maternal satisfaction, and decreases local anesthetic consumption compared with continuous epidural infusion (CEI) [5, 6].

Several studies have shown the benefits of PIEB compared with CEI in different types of surgery. Ueda et al. reported that PIEB using ropivacaine 0.75% resulted in a more extensive dermatomal spread as measured by loss of sensation compared with CEI following gynecologic surgery [10]. Kang et al. found that PIEB using bupivacaine 0.125% and morphine 0.005% resulted in lower numerical rating scale (NRS) scores for pain compared with CEI following total knee arthroplasty [11].

However, it remains unclear whether PIEB provides superior postoperative analgesia following open surgeries under combined general-epidural anesthesia. In this study, we compared the efficacy of postoperative analgesia using PIEB versus CEI with patient-controlled epidural analgesia (PCEA) at 3, 24, and 48 hours after surgery. We also investigated the number of PCEA boluses and the incidence of complications.

## 2. Materials and Methods

### 2.1. Patients and Study Protocol

The study was approved by the Human Research Ethics Committee of Tokushima University and was registered in a clinical trials data base (UMIN000018881). We conducted a randomized, double-blind clinical trial from June 2016 to August 2017. Written informed consent was obtained from all patients, and the study was conducted in accordance with the principals outlined in the Declaration of Helsinki.

**Figure 1:**
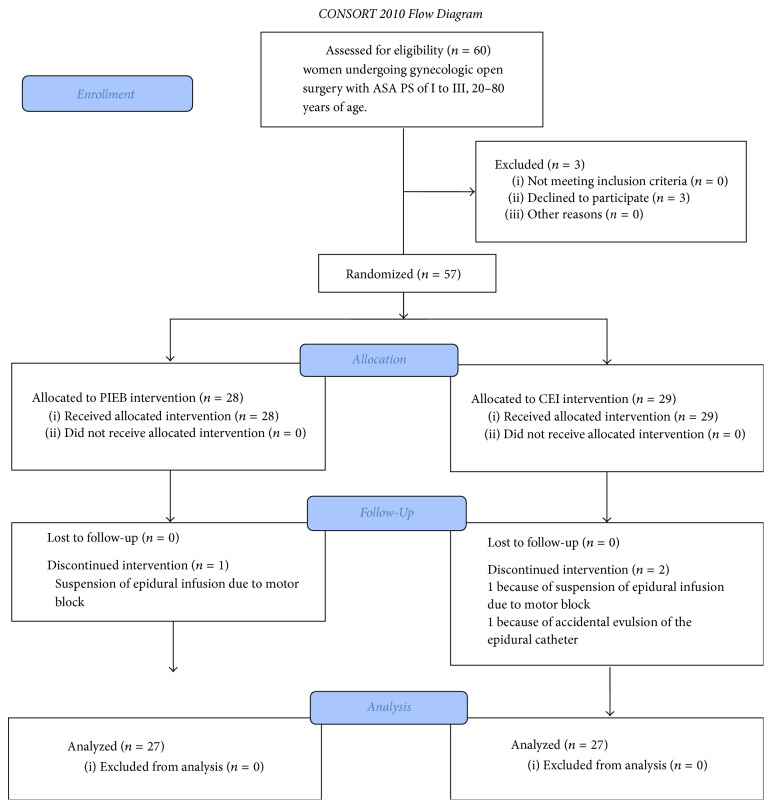
Consolidated Standards of Reporting Trials (CONSORT) flow diagram indicating patient disposition during the study. PIEB, programmed intermittent epidural bolus; CEI, continuous epidural infusion; ASA PS, American Society of Anesthesiologists Physical Status.

The recruited patients were women with myoma or gynecological cancer who were between 20 and 80 years of age, had an American Society of Anesthesiologists Physical Status (ASA PS) of I–III, and were scheduled to undergo open gynecological surgery that involved a lower midline skin incision that was less than 10 cm long or a vertical incision above the umbilicus that was less than 20 cm long. Patients were excluded from the study if they had any contraindication to epidural analgesia, their ASA PS score was ≥IV, they were <20 years old, they had received opioids, and/or consent was not obtained. The patients were randomized at a 1 : 1 ratio to receive either PIEB or CEI (Figure 1).

No patients were medicated prior to anesthesia induction. After entering the operating room, standard monitors were applied, including a blood pressure cuff, pulse oximetry probe, and ECG leads. An epidural catheter was inserted with the patient in the left lateral decubitus position at the T10-T11 or T11-T12 interspace before anesthesia induction. The epidural space was identified using loss of resistance to saline technique with an 18-gauge Tuohy epidural needle. A closed-end, multiorifice epidural catheter (Perifix FX, BBraun, Aesculap, Tuttlingen, Germany) was advanced 5 cm and 2 mL lidocaine of 1% was administered as a test dose. After epidural catheter placement, a blinded researcher who set up the epidural pump according to group allocation assigned the patient using computer-generated distribution (Quick-Calcs, GraphPad Inc., La Jolla, CA, USA) which is made by a statistician who was not involved in the clinical study. The subjects and other study personnel were blinded to group assignment and all observations and assessments were performed by a researcher blinded to the mode of drug administration.

General anesthesia was induced with propofol, remifentanil, and rocuronium and was maintained with sevoflurane or desflurane, remifentanil, and rocuronium. After tracheal intubation, all patients received an initial epidural loading dose of 6 mL ropivacaine 0.2% plus fentanyl 1.6 *μ*g/mL, and a CADD-Solis Ambulatory Infusion Pump (Smith Medical, St Paul, MN, USA) was connected.

**Figure 2:**
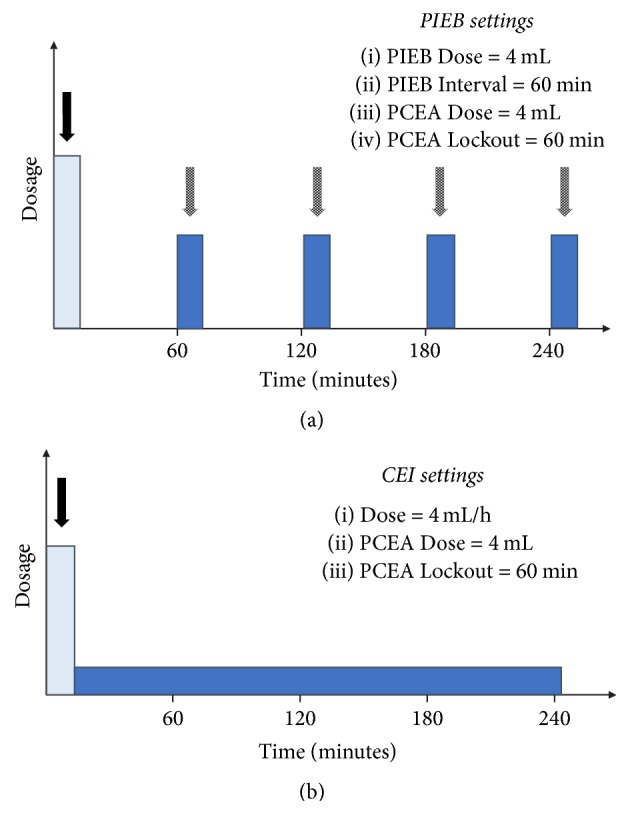
Pump settings in the (a) programmed intermittent epidural bolus (PIEB) and (b) continuous epidural infusion (CEI) groups. The first arrows in both figures show the initial bolus. Other arrows in (a) show the intermittent boluses.

In the PIEB group, the pump was programmed to deliver 4 mL ropivacaine 0.2% plus fentanyl 2 *μ*g/mL every hour beginning 1 hour after the epidural loading dose. In the CEI group, the pump was programmed to deliver ropivacaine 0.2% plus fentanyl 2 *μ*g/mL at a rate of 4 mL/h beginning immediately after the loading epidural dose (see Figure 2). The pump was programmed and set by an anesthesiologist who was not responsible for the anesthetic management of the patient. Flurbiprofen axetil 1 mg/kg was administered intravenously in both groups at the end of the surgery. The PCEA pump was programmed to deliver 4 mL/h of the same solution with a lockout interval of 1 hour and a maximum hourly volume of 12 mL. If patients requested additional analgesics, loxoprofen 60 mg was taken orally or pentazocine 15 mg was administered intravenously.

### 2.2. Measurements

All observations and assessments were performed by anesthesiologists who were not in charge of the anesthetic management of this study and who were blinded to the mode of drug administration. The primary outcome was the total dose of ropivacaine 40 hours after surgery. The secondary outcomes were the degree of postoperative pain (NRS pain scores), the number of PCEA boluses, the anesthesia range, the extent of sensory and motor block, the presence/absence of hypotension that required treatment, the presence/absence of postoperative nausea and vomiting (PONV), and the dose of loxoprofen and pentazocine 3, 24, and 48 hours after surgery. The highest postoperative pain between the prior assessment and the time of the assessment was evaluated by an 11-point NRS (0 = no pain to 10 = the worst pain imaginable). Extent of sensory blockade was assessed using loss of cold sensation. The number of administered PCEA requests was recorded by the CADD-Solis Medication System version 3 (Smith Medical, St Paul, MN, USA) and was obtained after patients completed the regimen. The degree of motor block was assessed in both lower extremities using the Breen modified Bromage score [12], whereby 1 is complete block (unable to move feet or knees), 2 is almost complete block (only able to move feet), 3 is partial block (just able to move knees), 4 is detectable weakness of hip flexion while supine (between scores 3 and 5), 5 is no detectable weakness of hip flexion while supine (full flexion of knees), and 6 is being able to stand and perform a partial knee bend. The severity of nausea was estimated by the nausea score (0 = absence of nausea; 1 = mild nausea; 2 = moderate nausea; 3 = severe nausea).

### 2.3. Statistical Analysis

The data from a study comparing PIEB and CEI in terms of the total dose of ropivacaine needed to achieve effective analgesia in labor were used for the power calculation. This calculation was based on mean (standard deviation) total ropivacaine doses of 104.7 (29.2) mg in the PIEB group and 124.2 (17.9) mg in the CEI group [3]. This analysis suggested that a study with 25 patients per group would have a power of 80% to detect a statistically significant difference in total ropivacaine dose at an alpha of 0.05. Patients who developed complications that prevented them from completing the trial (e.g., motor block and epidural catheter evulsion) were excluded from the perprotocol analysis. All data are presented as mean (standard deviation), median (25th–75th quartile range), or number of subjects. All analyses were performed by using the Statistical Package for Social Sciences (SPSS) software version 22 (IBM Corp, Armonk, New York, USA). The PIEB and CEI groups were compared by using the Chi-squared test or the Mann–Whitney *U* test. A *P* value of <0.05 was considered to indicate statistical significance.

## 3. Results

Sixty women were recruited to participate from July 2016 to August 2017. Of these, 3 patients declined to participate in the research and 57 patients were randomized to either the PIEB or CEI group. In the PIEB group, motor block on the right thigh with a modified Bromage scale score of 3 occurred in one patient. In the CEI group, sensory block on the anterior aspect of the right thigh and motor block on the right thigh with a modified Bromage scale score of 3 occurred in one patient. Accidental evulsion of the epidural catheter occurred in one patient. In all, 54 patients completed the trial. Patient demographics are shown in Table 1.

**Figure 3:**
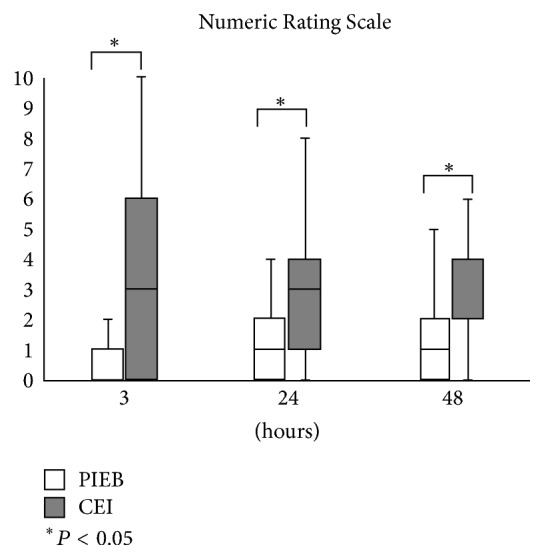
Comparison of the programmed intermittent epidural bolus (PIEB) and continuous epidural infusion (CEI) groups in terms of numeric rating scale (NRS) of pain 3, 24, and 48 hours after surgery. The data are shown as medians (lines in the boxes), 25th and 75th quartiles (bottom and top of the boxes, resp.), and the lower and upper outlier thresholds (the ends of the whiskers on the boxes). The lower outlier threshold was the 25th percentile minus 1.5 × the interquartile range (IQR; 75th quartile minus 25th quartile) while the upper outlier threshold was the 75th percentile plus 1.5 × the IQR. ^*∗*^Significant (*P* < 0.05) differences between the PIEB and CEI groups, as indicated by Mann–Whitney *U* tests.

There was significant difference in the total dose of ropivacaine 40 hours after surgery (mean (standard deviation): 155.38 (4.55) versus 159.73 (7.87) mL, *P* = 0.016). NRS scores were significantly different between the two groups at 3 hours (median [IQR], 0 [0–0.5] versus 3 [0–5.5], *P* = 0.002), 24 hours (1 [0–2] versus 3 [1–4], *P* = 0.003), and 48 hours after surgery (median [IQR], 1 [0–2] versus 2 [2–3.5], *P* = 0.002) (Figure 3). The number of administered PCEA boluses was significantly different between the two groups at 3 hours (median [IQR], 0 [0-0] versus 0 [0-1], *P* = 0.004) and 24 hours (median [IQR], 0 [0-0] versus 0 [0–2], *P* = 0.045). The total number of PCEA boluses was significantly different between the two groups (median [IQR], 0 [0-0] versus 1 [0–4], *P* = 0.007). The dose of oral administration of loxoprofen was significantly different between the two groups at 48 hours (median [IQR], 0 [0–60] versus 60 [30–120], *P* = 0.036). The total dose of loxoprofen was significantly different between the two groups (median [IQR], 60 [0–150] versus 120 [60–180], *P* = 0.047, resp.) (Table 2).

No significant between-group difference was observed in any of the other parameters evaluated (Table 3).

## 4. Discussion

In this study, we compared the efficacy of PIEB with that of CEI for postoperative analgesia following open gynecologic surgery. PIEB with 4 mL of ropivacaine 0.2% plus fentanyl 2 *μ*g/mL every hour resulted in lower dose of ropivacaine 40 hours after surgery and NRS scores at 3, 24, and 48 hours after surgery compared with CEI using the same hourly volume of the same solution. Also, the total number of PCEA boluses was smaller in the PIEB group. Moreover, there was no difference in the incidence of complications between the two groups. These results suggest that PIEB can safely provide better postoperative analgesia than CEI after open gynecologic surgery.

The efficacy of epidural analgesia requires adequate spread of the anesthetic solution within the epidural space to produce sensory blockade of the appropriate dermatomes [6]. It has been demonstrated that distribution of solution through an epidural catheter is not uniform. The most uniform spread occurs with large volumes and correspondingly high injectate pressures near the site of injection [13]. An in vitro study using the computer-aided design and drafting (CADD) pump showed that peak pressure was directly associated with the delivery speed of the solution [14]. Solutions injected into the epidural space also tend to spread more evenly when injected as a bolus, which likely explains why more effective analgesia was achieved in the PIEB group.

Many studies have sought to determine the optimal PIEB regimen and pump settings [2–11]. For labor analgesia, Wong et al. demonstrated that extending the programmed intermittent bolus interval from 15 to 60 minutes and increasing the volume from 2.5 to 10 mL decreased bupivacaine consumption without decreasing patient comfort or satisfaction [15]. Kanczuk et al. found that the optimal time interval between boluses with PIEB using 10 mL of bupivacaine 0.0625% plus fentanyl 2 *μ*g/mL to achieve effective analgesia in 90% of women during the first stage of labor without breakthrough pain was approximately 40 minutes [16]. The bolus volume and lockout period vary significantly among PIEB studies; therefore further studies are needed to determine the optimal regimen.

In combined general-epidural aesthesia, Ueda et al. reported that PIEB using ropivacaine 0.75% at a dose of 1 mL every 20 minutes resulted in a more extensive dermatomal spread, as measured by loss of sensation, in gynecologic surgery compared with CEI using the same solution at a dose of 3 mL/h [10]. Kang et al. found that PIEB using 3 mL of bupivacaine 0.125% plus morphine 0.005% every hour resulted in lower NRS scores compared with CEI after total knee arthroplasty [11]. In our study, we chose to use 4 mL/hour of ropivacaine 0.2% plus fentanyl 2 *μ*g/mL as the epidural analgesic regimen during and after gynecological surgery on the basis of these studies and a study on CEI with 0.2% ropivacaine/hour in abdominal surgery [17]. Ropivacaine was chosen rather than bupivacaine because it is less toxic and generates less motor block when given by epidural infusion [18]. The flow rate was set to a slow speed to decrease the complications of epidural anesthesia. Our regimen of PIEB did not require many PCEA doses or rescue analgesics, which is consistent with previous studies [5, 6]. PIEB may be therefore be an effective technique for both combined general-epidural anesthesia and labor analgesia.

The two groups did not differ in terms of PONV at any time point. This may reflect the fact that fentanyl was also used for pain relief and mitigated the tendency of ropivacaine to induce PONV.

Our study does have some limitations. First, we only included patients undergoing open gynecological surgery under combined general-epidural anesthesia. Further studies are therefore needed to confirm the efficacy of PIEB with different types of surgeries in different regions of the body. Another limitation of our study was that all study participants were women less than 80 years old. Because pain perception differs with gender and age [19, 20], epidural anesthesia settings may need to be adjusted for each patient.

## 5. Conclusions

PIEB reduced the total ropivacaine dose 40 hours after surgery. NRS scores were also significantly lower in patients who received PIEB compared with those who received CEI at 3, 24, and 48 hours postoperatively. PIEB therefore provides superior postoperative analgesia to CEI following open gynecologic surgery under combined general-epidural anesthesia. PIEB is one of the effective analgesic techniques for postoperative pain. To optimize the PIEB regimen, future studies are needed.

## Figures and Tables

**Table 1:** Baseline demographic and clinical characteristics of the programmed intermittent epidural bolus (PIEB) and continuous epidural infusion (CEI) groups.

Variable	PIEB group	CEI group	*P* value
	(*n* = 28)	(*n* = 29)	
Age, year			
30–39	3	5	0.478
40–49	7	9	0.612
50–59	7	5	0.473
60–69	8	6	0.490
70–79	3	4	0.723
Mean ± SD	54 ± 11	53 ± 12	0.669
Height, cm	156 ± 5	158 ± 7	0.196
Weight, kg	57 ± 12	60 ± 11	0.382
BMI, kg/m^2^	23 ± 5	24 ± 5	0.572
ASA PS I/II/III	7/20/1	12/16/1	0.418
Duration of anesthesia, min	294 ± 137	299 ± 124	0.888
Duration of surgery, min	251 ± 134	251 ± 125	0.995
Type of surgery			
Myomectomy	2	4	0.413
TAH	2	3	0.669
BSO	4	2	0.363
TAH + BSO	7	6	0.698
TAH + BSO + pelvic lymph node dissection	5	4	0.674
TAH + BSO + omentectomy + pelvic para-aortic lymph node dissection	8	10	0.631
Fluid volume, mL	2563 ± 1549	2441 ± 1214	0.747
Length of hospital stay, days	12 ± 5	13 ± 7	0.589

The data are presented as mean ± SD or number of patients; ASA PS: American Society of Anesthesiologists Physical Status; BMI: body mass index; BSO: bilateral salpingo-oophorectomy; CEI: continuous epidural infusion; PIEB: programmed intermittent epidural bolus; TAH: total abdominal hysterectomy.

**Table 2:** Postoperative medication used by the programmed intermittent epidural bolus (PIEB) and continuous epidural infusion (CEI) groups (perprotocol analysis).

Variable	PIEB group	CEI group	*P* value
	(*n* = 27)	(*n* = 27)	
Number of PCEA doses			
3 hours	2	13	0.004
24 hours	13	33	0.045
48 hours	4	12	0.362
Total	17	60	0.007
Loxoprofen, mg, median [25th–75th quartiles]			
3 hours	0 [0-0]	0 [0-0]	1.000
24 hours	0 [0–60]	60 [0–60]	0.431
48 hours	0 [0–60]	60 [30–120]	0.036
Total	60 [0–150]	120 [60–180]	0.047
Pentazocine, mg, median [25th–75th quartiles]			
3 hours	0 [0-0]	0 [0-0]	1.000
24 hours	0 [0-0]	0 [0-0]	0.317
48 hours	0 [0-0]	0 [0-0]	1.000
Total	0 [0-0]	0 [0-0]	0.624

The data are presented as median (25th–75th quartiles) number of administered doses or dose; CEI: continuous epidural infusion; PCEA: patient-controlled epidural analgesia; PIEB: programmed intermittent epidural bolus.

**Table 3:** Postoperative complications in the programmed intermittent epidural bolus (PIEB) and continuous epidural infusion (CEI) groups (intention-to-treat analysis).

Variable	PIEB group	CEI group	*P* value
	(*n* = 28)	(*n* = 29)	
3 hours			
Postoperative nausea and/or vomiting	14	19	0.236
Sensory and/or motor block	1	1	0.980
Hypotension	0	0	1.000
24 hours			
Postoperative nausea and/or vomiting	8	13	0.200
Sensory and/or motor block	0	0	1.000
Hypotension	0	0	1.000
48 hours			
Postoperative nausea and/or vomiting	3	9	0.050
Sensory and/or motor block	0	0	1.000
Hypotension	0	0	1.000

All data are presented as total numbers; CEI: continuous epidural infusion; PIEB: programmed intermittent epidural bolus.

## Data Availability

Requests for data will be considered by the corresponding author.
